# Overexpression of miR-340 inhibits cell proliferation and induces apoptosis of human bladder cancer via targeting Glut-1

**DOI:** 10.1186/s12894-021-00935-z

**Published:** 2021-12-03

**Authors:** Gang Xu, Shouhua Pan, Zhirong Zhu, Junlong Li

**Affiliations:** Department of Urology, Shaoxing People’s Hospital (Shaoxing Hospital, Zhejiang University School of Medicine), No. 568, Zhongxing North Road, Shaoxing, 312000 Zhejiang China

**Keywords:** Bladder cancer, miR-340, Glut-1, Proliferation, Apoptosis

## Abstract

**Background:**

Bladder cancer (BC) has high mortality due to distant metastasis. Previous works suggested that microRNA (miRNA)-340 is a critical regulator for the development and progression of various cancers. The specific biological function of miR-340 in BC is little known.

**Methods:**

In the present study, RT-qPCR was performed to measure the expression of miR-340 in paired BC tissues and adjacent non-tumor tissues. Next, the target gene of miR-340 was identified using dual-luciferase reporter assay and its level was also tested in tissues. Moreover, cell proliferation and apoptosis were analyzed by CCK-8 and flow cytometry. Finally, the expression of PCNA, Bax was detected by RT-qPCR and western blotting, as well as PI3K/AKT signaling measured by western blotting.

**Result:**

The results demonstrated that miR-340 expression was downregulated and its target Glut-1 level was upregulated in BC tissues. Functionally, overexpression of miR-340 suppressed the proliferation and induced apoptosis in BC cells, while Glut-1 reversed the suppression of proliferation or induction of apoptosis induced by miR-340. Additionally, miR-340 repressed PCNA, p-PI3K and p-AKT levels but enhanced Bax level, while Glut-1 rescued the effects.

**Conclusion:**

In conclusion, miR-340 functions as a tumor suppressor of BC, which inhibited proliferation and induced apoptosis by targeting Glut-1 partly through regulating PCNA, Bax expression and PI3K/AKT pathway. This study suggested that miR-340 is a potential target for the treatment of BC.

**Supplementary Information:**

The online version contains supplementary material available at 10.1186/s12894-021-00935-z.

## Introduction

Bladder cancer (BC) is the ninth most general malignancy in the worldwide with the thirteenth deadliest [1]. BC can be divided into most common type: transitional cell carcinoma (more than 90%) and less common type: squamous cell carcinoma (5%) and adenocarcinoma (less than 2%) [2]. The incidence of this disease is higher in males than females because of the high prevalence of smoking [3]. The other risk factors for BC include age, body weight, lifestyle, and heredity [4]. Most patients with advanced disease develop metastasis including lymph nodes, bone, lung, liver, and peritoneum, and the 5-year survival rate is 96% for preinvasive carcinoma, but only 5% in distant metastasis unfortunately [5]. Although diagnosis and treatment for BC has little progress in recent 30 years [6], the emergence of immunotherapy, biomarkers and advanced imaging is hopeful for improving treatment effects for patients with BC [7]. To data, only a few of BC biomarkers are approved for clinical use [8], so it is urgent to find more meaningful targets for early screening and treatment of BC.

MicroRNAs (miRNAs) are a class of 20–30 nucleotides (nt), noncoding RNAs that regulate endogenous genomes expression and function [9]. They regulate gene expression through targeting mRNA cleavage or translational repression, and mediate cell proliferation, apoptosis, differentiation and metabolism [10]. Aberrantly expressed miRNAs are usually observed in clinical tissues, urine and blood of patients with BC, which is associated with tumor stage, recurrence and chemosensitivity [11]. These abnormal miRNAs play important role in BC development, progression and metastasis, as well as diagnostic and prognostic biomarkers [12]. MiR-340 is a multifunctional miRNA, which is involved in several diseases’ programs, such as activity-based anorexia [13], Parkinson's disease [14], endometrial fibrosis [15] and human cancers [16–18]. For example, in pediatric patients with osteosarcoma, Cai et al. reported that miR-340-low/ROCK1-high mRNA is associated with poorer OS and PFS, which is an independent prognostic factor according to multi-variants analysis [19]. Danilo Fiore et al. reported that miR-340 could predict glioblastoma survival and modulates key cancer hallmarks through down-regulation of NRAS in glioblastoma [20]. Huang et al. reported that MiR-340 could inhibit prostate cancer cell proliferation and metastasis by targeting the MDM2-p53 pathway [21]. MicroRNA-340 could induce apoptosis and inhibit metastasis of ovarian cancer cells by inactivation of NF-κB1 [22]. MicroRNA-340 could inhibit the growth and invasion of angiosarcoma cells by targeting SIRT7 [23]. Loss of miR-340 function could enhanced lactate secretion and glucose uptake rate [16]. Jian et al. suggested that knock-down of Glut-1 in osteosarcoma cell could inhibited cell growth in vitro [7]. Xu and coworkers reported that miR-340 could accelerate tumor cell glycolysis and promote cell growth and proliferation via increasing the expression of Glut-1 in oral squamous cell carcinoma [24]. Though promising, most miR-340 related studies are still in early stage currently and it is little known about the role of miR-340 in BC. In the present study, we studied the relationship between miR-340 expression and Glut-1 in BC tissues cells.

## Materials and methods

### Tissue samples collection

BC tissues and adjacent normal tissues were obtained after resection from 30 cases of patients who underwent surgery at Shaoxing People’s Hospital. These samples were immediately frozen in liquid nitrogen and stored at -80˚C refrigerator. Written informed consent was provided by all patients participated in our study. This study was approved by the Ethic Committee of Shaoxing People’s Hospital.

### Cell culture

Human BC cell line T24 was purchased from American Type Culture Collection (ATCC; Manassas, VA, USA). T24 cells were cultured in RPMI 1640 medium supplemented with 10% fetal bovine serum (FBS) and 1% penicillin–streptomycin (All reagents were acquired from Hyclone, South Logan, UT, USA). The cultured condition of cells was maintained at 37˚C in a humidified atmosphere containing 5% CO_2_.

### Dual-luciferase reporter assay

The target of miR-340 was predicted by bioinformatics online tool TargetScan Human 7.2 (http://www.targetscan.org/vert_72/) and verified by dual-luciferase reporter assay. MiR-340 mimics and its matched negative control (miR-NC mimics) were purchased from GeneCopoeia (Rockville, MD, USA). pGL3 vector (Promega, Madison, WI, USA) containing wild-type (WT) binding site of Glut-1 or mutant type of Glut-1 was co-transfected with miR-340 mimics or miR-NC mimics into T24 cells using Lipofectamine 2000 (Invitrogen, Carlsbad, CA, USA). 48 h later, Luc-Pair Duo-Luciferase Assay Kit was used to detect Firefly luciferase and Renilla luciferase activities (GeneCopoeia). Renilla luciferase activity was performed as the endogenous control.

### Transient transfection

The coding sequence of Glut-1 was cloned into pcDNA3.1 plasmid, termed as pcDNA3.1-Glut-1. T24 cells were seeded into 6-well plates at the density of 2 × 10^5^ cells/well. After cells growth to 80% confluence, miR-340 mimics, miR-NC mimics, pcDNA3.1 and pcDNA3.1-Glut-1 were transfected into T24 cells using Lipofectamine 2000 (Invitrogen) following manufacturer’s protocol. After transfection of 48 h, the cells were collected for further study.

### RT-qPCR

Total RNA was isolated from tissues and T24 cells using RNAsimple Total RNA Kit (Tiangen, Beijing, China). The purity of RNA was detected at OD260/OD280, and the integrality was visualized by agarose gel electrophoresis. The reverse transcription was determined by FastQuant RT Super Mix (Tiangen). Then SuperReal PreMix Plus (SYBR Green) (Tiangen) was used to examine the miR-340, Glut-1, PCNA and Bax expression through ABI PRISM 7700 Real-time PCR system (Applied Biosystems, Foster City, CA, USA) with the reaction condition as follows: 95˚C for 15 min, 40 cycles of 95˚C for 10 s and 60˚C for 32 s. The expression of U6 and GAPDH was detected as the normalization for miR-340 and other mRNAs, respectively. The fold change of relative expression was calculated by using 2^−△△Ct^ method.

### Western blotting

Total protein was extracted from tissues and cells using RIPA lysis buffer (Solarbio, Beijing, China) and the concentration was estimated by BCA Protein Assay Kit (Solarbio). Equal amount of extracted protein samples was loaded on 10% SDS-PAGE and subsequently transferred to PVDF membrane (Millipore, Billerica, MA, USA). Afterwards, the membranes were blocked with TBST mixed with 5% non-fat milk, incubated with primary antibodies overnight at 4˚C, and then incubated with secondary antibodies for 1 h at room temperature. All primary antibodies including anti-Glut-1, anti-PCNA, anti-Bax, anti-PI3K, anti-p-PI3K, anti-AKT, anti-p-AKT, anti-GAPDH and secondary antibody were purchased from Abcam (Cambridge, MA, USA). GAPDH was used as the loading control. Finally, each protein signals were visualized using ECL chemiluminescence detection kit (Jining Shiye, Shanghai, China).

### Cell proliferation and apoptosis analysis

After cells were trandfected with miR-340 mimics, miR-NC mimics and pcDNA3.1-Glut-1, cell proliferation and apoptosis were assessed by Cell Counting Kit-8 (CCK-8; Dojindo, Kumamoto, Japan) and Annexin V-APC/7-AAD Apoptosis Detection Kit (MultiSciences, Hangzhou, China). Briefly, cell proliferation was detected at cells incubated at 37˚C with 5% CO_2_ for 0 h, 12 h, 24 h and 48 h. Each well was added with 10 μl CCK-8 solution and continued to incubate for 2 h. A microplate reader was used to measure the OD value at the wavelength of 450 nm. For cell apoptosis, transfected cells were washed twice with PBS and resuspended with 100 μl binding buffer. After that, 5 μl Annexin V-APC and 10 μl 7-AAD staining solution were added to incubated with cells for 15 min at room temperature. After adding 380 μl binding buffer, the samples were analyzed using flow cytometry.

### Data analysis

All data was analyzed by Graphpad Prism 6.0 La Jolla, CA, USA) from at least three independent experiments, and showed as mean ± SEM. The differences were assessed by Student’s t-test and one-way ANOVA between two groups or multiple groups. The statistical significance was defined by P < 0.05.

## Results

### Downregulation of miR-340 is found in BC tissues

**Fig. 1:**
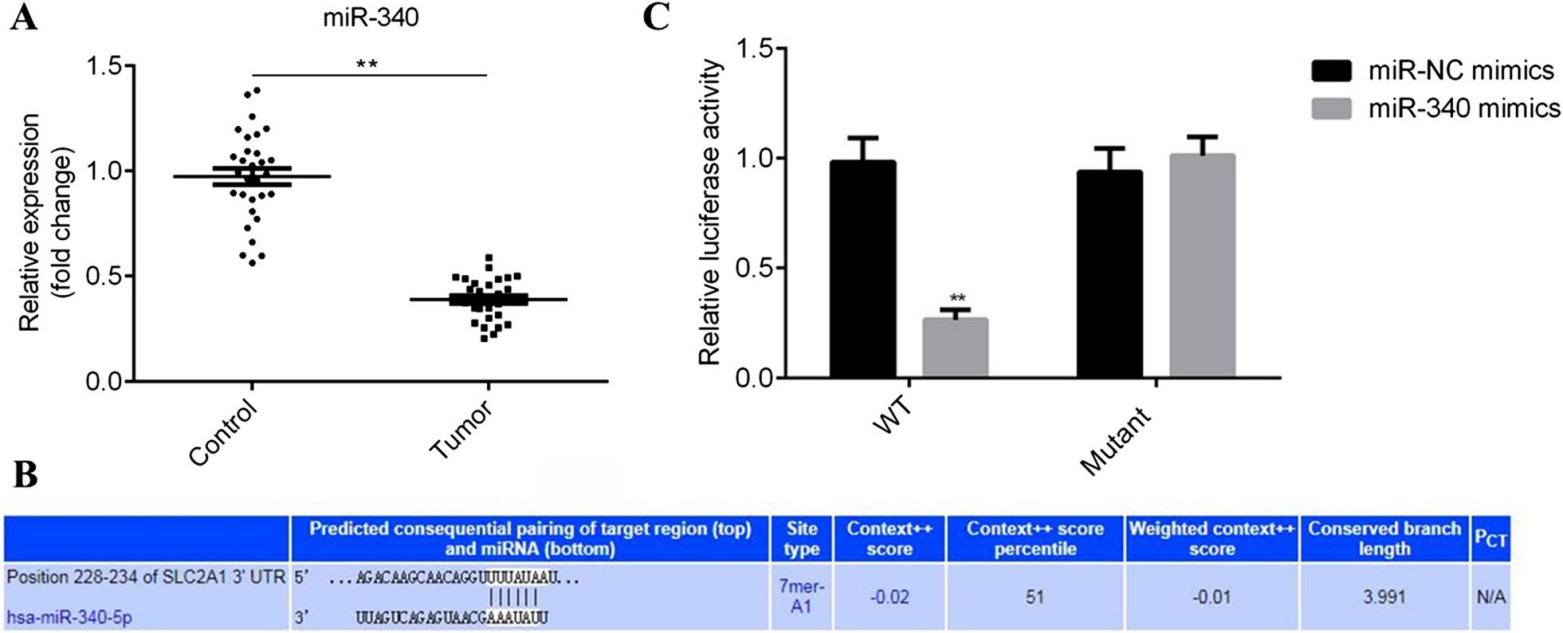
Glut-1 is a potential target of miR-340. **A** MiR-340 expression is downregulated in BC tissues, which was detected by RT-qPCR in both tumor tissues and corresponding adjacent normal tissues collected a total of 30 cases of patients with BC. **P < 0.01. **B** The binding site between miR-340 and 3’UTR of Glut-1 was predicted by TargetScan. **C** The relative luciferase reporter assay was measured after T24 cells co-transfection of miR-340 mimics or miR-NC mimics together with WT-Glut-1 or mutant-Glut-1with dual-luciferase reporter assay. **P < 0.01

To investigate the role of miR-340 in BC, 30 pairs of tumor tissues and matched non-tumor tissues were collected from patients with BC. The corresponding baseline characteristics was shown in Additional file 14: Table S1. The results of RT-qPCR showed that the expression of miR-340 was decreased in BC tissues, compared with adjacent tissues (P < 0.01; Fig. 1A).

### MiR-340 targets Glut-1 in BC cells

Through the bioinformatics online tool TargetScan Human 7.2, Glut-1 was screened as a potential target of miR-340. Glut-1 is an integral membrane protein providing glucose pass through plasma membrane down its concentration gradient. As shown in Fig. 1B, miR-340 could bind to the 3’-UTR of Glut-1 at the position of 228–234 nt, but not the mutant position of 3’UTR. Moreover, the results of dual-luciferase reporter assay demonstrated that the relative luciferase activity was weakened by overexpression of miR-340 when cells cotransfected with WT type of Glut-1 3’UTR, compared with miR-NC mimics (P < 0.01), which was not affected when Glut-1 3’UTR was mutant (Fig. 1C).

### The expression of Glut-1 is increased in BC tissues and negatively regulated by miR-340 in BC cells

**Fig. 2:**
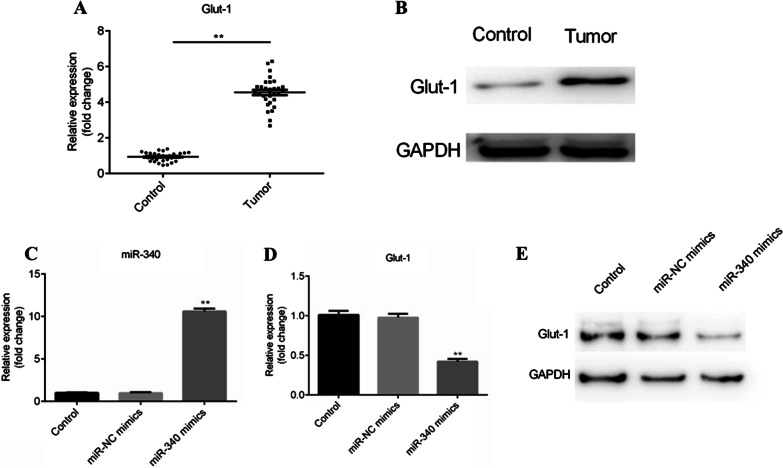
The level of Glut-1 was found to be upregulated in BC tissues and negatively regulated by miR-340 overexpression. **A** and **B** The mRNA and protein expression of Glut-1 was tested in 30 pairs of BC tissues and adjacent normal tissues by RT-qPCR and western blotting, respectively. Full length blots/gels are presented in Additional file 1: Fig. S1–Additional file 2: Fig. S2. **C** The transfection efficiency was examined by RT-qPCR after T24 cells transfection of miR-340 mimics and miR-NC mimics for 48 h. **D** and **E** The mRNA and protein levels of Glut-1 were detected using RT-qPCR and western blotting after T24 cells transfection of miR-340 mimics and miR-NC mimics. **P < 0.01. Full length blots/gels are presented in Additional file 3: Fig. S3–Additional file 4: Fig. S4

Then we detected the expression of Glut-1 in BC tissues by RT-qPCR and western blotting. The mRNA level of Glut-1 is upregulated in tumor tissues, compared with corresponding para-carcinoma tissues (P < 0.01; Fig. 2A). Similarly, Glut-1 protein level was also elevated in BC tissues (Fig. 2B). In addition, after T24 teansfected with miR-340 mimics and miR-NC mimics, the level of miR-340 is significantly increased in miR-340 mimics group, compared with miR-NC mimics (P < 0.01; Fig. 2C), meanwhile the expression of Glut-1 was downregulated in miR-340 mimics group (P < 0.01; Fig. 2D, E).

### Overexpression of miR-340 inhibits BC cell proliferation and enhances apoptosis through targeting Glut-1 in vitro

**Fig. 3:**
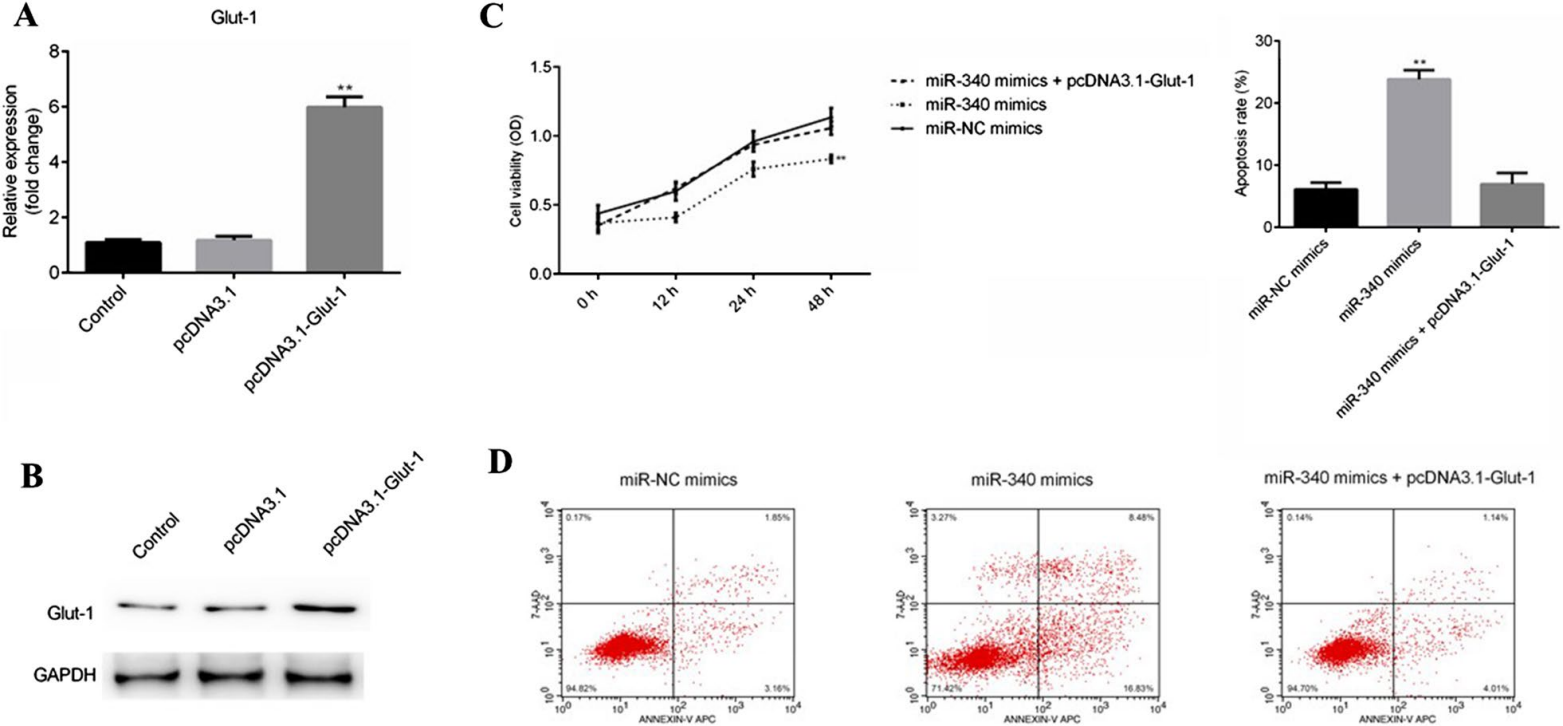
MiR-340 targets Glut-1 to suppress the proliferation and promote apoptosis in BC cell line. **A** and **B** The transfection efficiency was tested after T24 cells transfected with pcDNA3.1 and pcDNA3.1-Glut-1 using RT-qPCR and western blotting. Full length blots/gels are presented in Additional file 5: Fig. S5–Additional file 6: Fig. S6. **C** Cell proliferation was measured at transfected cells incubated for 0, 12, 24 and 48 h using CCK-8 assay. **D** Cell apoptosis was tested by flow cytometry after transfected cells incubated for 24 h, and cell apoptosis rate was quantified. **P < 0.01

To explore the function role of miR-340 and Glut-1, pcDNA3.1-Glut-1 and pcDNA3.1 were also transfected into T24 cells, and transfection efficiency was examined. Glut-1 level was increased in pcDNA3.1-Glut-1 group, compared with pcDNA3.1 group (P < 0.01; Fig. 3A, B). Importantly, the results of CCK-8 demonstrated that cell proliferation was suppressed after 48 h by overexpression of miR-340, compared with miR-NC mimics (P < 0.01), which was reversed by restoration of Glut-1 (Fig. 3C). Inversely, the data of flow cytometry indicated that cell apoptosis was induced by miR-340 mimics (P < 0.01), which was abolished by pcDNA3.1-Glut-1 (Fig. 3D).

### MiR-340 regulates PCNA, Bax expression and PI3K/AKT signaling pathway through targeting Glut-1

**Fig. 4:**
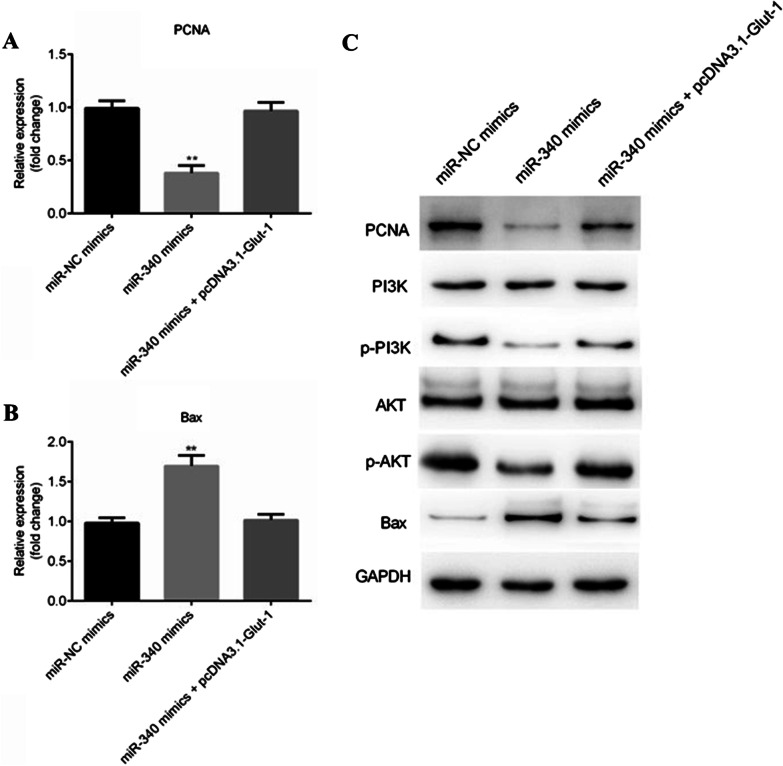
Upregulation of miR-340 targets Glut-1 to regulate PCNA and Bax levels, as well as PI3K/AKT pathway. **A** and **B** The expression of PCNA and Bcl-2 at mRNA level was detected by RT-qPCR. **C** The protein levels of PCNA, Bcl-2 and PI3K/AKT pathway related factors such as PI3K, p-PI3K, AKT and p-AKT were detected by western blotting. GAPDH was used as the internal control. **P < 0.01. Full length blots/gels are presented in Additional file 7: Fig S7–Additional file 13: Fig. S13

Finally, the expression of PCNA and Bax was tested by RT-qPCR and western blotting. As shown in Fig. 4A, B, overexpression of miR-340 repressed PCNA level but enhanced Bax level (P < 0.01), while Glut-1 reversed the inhibitory effects on PCNA and the enhancement on Bax induced by miR-340. Similarly, the changes of protein expression of PCNA and Bax were consistent with those of mRNA (Fig. 4C). In addition, overexpression of mir-340 resulted in a decreased phosphorylation of PI3K and AKT, which was rescued by Glut-1 restoration, while total PI3K and AKT expression was not affected by miR-340 and Glut-1 (Fig. 4C).

## Discussion

MiR-340 is commonly functions as a tumor suppressor in human cancers, which regulates cellular processes such as proliferation, apoptosis, migration and invasion. For example, miR-340 reduced cell viability and proliferation, and induced cell apoptosis of gastric cancer [25]. Additionally, overexpression of miR-340 suppressed cell proliferation, migration and invasion through targeting NT5E of gallbladder cancer [26]. Moreover, miR-340 could block hepatocellular carcinoma cell proliferation and promote apoptosis via regulating DcR3 expression [27]. Similarly, in the present study, miR-340 expression was lower in BC tissues than that in matched normal tissues, and overexpression of miR-340 inhibited T24 cell proliferation and enhanced apoptosis, which suggested that miR-340 also functions as a tumor suppressor in BC.

This study also revealed the underlying molecular mechanism. Glut-1 is a potential target of miR-340, which was predicted by TargetScan and verified by dual-luciferase reporter assay. Glucose transporter (Glut) proteins are the first rate-limiting step for glucose metabolism in mammalian cells, and overexpression of Glut-1 is associated with increased glucose transport, thus accelerating metabolism and accelerating glucose uptake and promoting malignant cell growth [28]. Recent years, Glut-1 is reported to be highly expressed in several human cancer types including but not limited to nasopharyngeal cancer [29], gastric cancer [30] and breast cancer [31]. Also, overexpressed of Glut-1 in malignant predicts poor prognosis [32–34]. In BC, the upregulation of Glut-1 is an independent prognostic factor and related to a low survival rate [35, 36]. However, the biological function of Glut-1 in BC is still unknown. In the present study, we confirmed that Glut-1 level was increased in BC tissues. Moreover, overexpression of miR-340 induced the downregulation of Glut-1, and restoration of Glut-1 reversed the inhibition of proliferation and the promotion of apoptosis induced by miR-340 in BC cells. These results suggested that miR-340 has suppressive effects on BC through targeting Glut-1.

Accumulating evidence shows that numerous miRNAs are related to molecular and cellular mechanism of BC, besides miR-340. Previous works suggested that downregulation of miR-145 and upregulation of miR-21 in bladder cancer. Prognostic relationships were seen with miRs-133b/129/518c. High-grade tumors were characterized by upregulation of miR-21, which probably reflected a p53-targeting role. Prognostic relationships were seen with miRs-133b/129/518c. MiRNAs appear to be important modulators of urologic cancer.

In this study, PCNA, p-PI3K and p-AKT levels were repressed, and Bax level was elevated by miR-340 but reversed by Glut-1, which suggested that miR-340 has suppressive effect on proliferation and promoting effect on apoptosis through decreasing PCNA expression, increasing Bax expression and inactivating PI3K/AKT signaling pathway. PCNA, a eukaryotic replication accessory factor, plays a critical role in cell cycle, apoptosis and proliferation in many types of human cancers [37]. Bax is a pro-apoptotic member of the Bcl-2 family, which is important for tumor therapy by regulating its expression [38]. The PI3K/AKT pathway is a commonly altered signaling network in cancers, and inhibition of AKT activity contributes to anticancer therapy [39]. These results suggested that PCNA, Bax and PI3K/AKT pathway associated with the cell growth and cell apoptosis, even though the potential mechanism between miR-340 and PCNA, Bax or PI3K/AKT pathway is still unclear. In view of the intrinsic property of pre-clinical study, several limitations existed, such as the small number of BC tissue samples and lack of analysis per grade, tumor stage and cancer type (invasiveness vs non-invasiveness) in the present work. Lack of animal experiment is another limitation, which should be investigated in next step.

In conclusion, our study revealed that miR-340 in BC functions as a tumor suppressor. Overexpression of miR-340 resulted in the decreased expression of target gene Glut-1, subsequently inhibited the proliferation and induced apoptosis partly through repressing PCNA, promoted Bcl-2 expression and inactivating PI3K/AKT signaling pathway of BC. This study revealed that miR-340 is a potential therapeutic target for the treatment of BC.

## Supplementary Information

**Additional file 1. Fig. S1**: GAPDH.

**Additional file 2. Fig. S2**: Glut-1.

**Additional file 3. Fig. S3**: GAPDH.

**Additional file 4. Fig. S4**: Glut-1.

**Additional file 5. Fig. S5**: GAPDH.

**Additional file 6. Fig. S6**: Glut-1.

**Additional file 7. Fig. S7**: GAPDH.

**Additional file 8. Fig. S8**: Bax.

**Additional file 9. Fig. S9**: p-AKT.

**Additional file 10. Fig. S10**: AKT.

**Additional file 11. Fig. S11**: p-PI3K.

**Additional file 12. Fig. S12**: PI3K.

**Additional file 13. Fig. S13**: PCNA.

**Additional file 14. Table S1**: Baseline characteristics.

## Data Availability

The datasets used and/or analyzed during the present study are available from the corresponding author on reasonable request.
