# Supplementary figures and images for: Overexpression of miR-340 inhibits cell proliferation and induces apoptosis of human bladder cancer via targeting Glut-1
Source: BMC Urol. 2021 Dec 3;21:168. doi: 10.1186/s12894-021-00935-z (PMC8641194; doi:10.1186/s12894-021-00935-z)

Figure S1. GAPDH

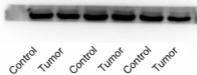

Supplement: Supplementary file 1 — Additional file 1. Fig. S1: GAPDH. [file 12894_2021_935_MOESM1_ESM.pdf]

Figure S2 Glut-1

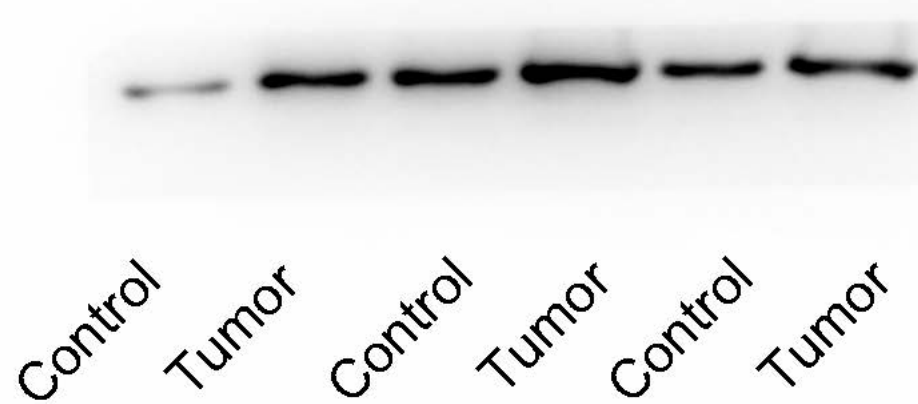

Supplement: Supplementary file 2 — Additional file 2. Fig. S2: Glut-1. [file 12894_2021_935_MOESM2_ESM.pdf]

Figure S3. GAPDH

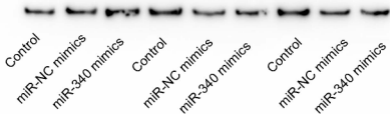

Supplement: Supplementary file 3 — Additional file 3. Fig. S3: GAPDH. [file 12894_2021_935_MOESM3_ESM.pdf]

Figure S4. Glut-1

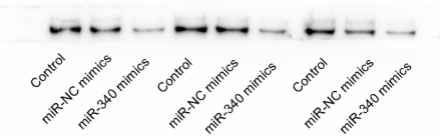

Supplement: Supplementary file 4 — Additional file 4. Fig. S4: Glut-1. [file 12894_2021_935_MOESM4_ESM.pdf]

Figure S5. GAPDH

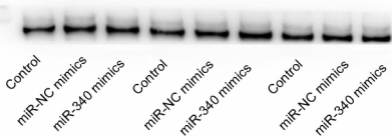

Supplement: Supplementary file 5 — Additional file 5. Fig. S5: GAPDH. [file 12894_2021_935_MOESM5_ESM.pdf]

Figure S6. Glut-1

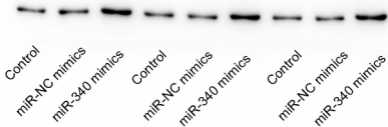

Supplement: Supplementary file 6 — Additional file 6. Fig. S6: Glut-1. [file 12894_2021_935_MOESM6_ESM.pdf]

Figure S7. GAPDH

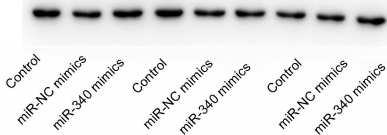

Supplement: Supplementary file 7 — Additional file 7. Fig. S7: GAPDH. [file 12894_2021_935_MOESM7_ESM.pdf]

Figure S8. Bax

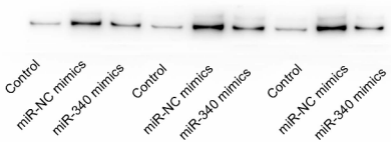

Supplement: Supplementary file 8 — Additional file 8. Fig. S8: Bax. [file 12894_2021_935_MOESM8_ESM.pdf]

Figure S9. p-AKT

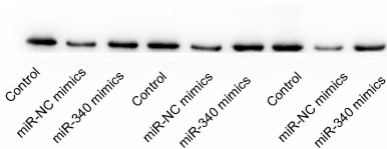

Supplement: Supplementary file 9 — Additional file 9. Fig. S9: p-AKT. [file 12894_2021_935_MOESM9_ESM.pdf]

Figure S10. AKT

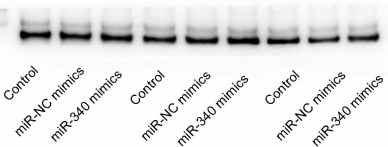

Supplement: Supplementary file 10 — Additional file 10. Fig. S10: AKT. [file 12894_2021_935_MOESM10_ESM.pdf]

Figure S11 p-PI3K

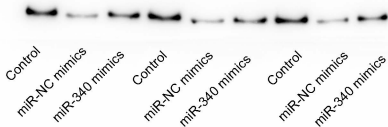

Supplement: Supplementary file 11 — Additional file 11. Fig. S11: p-PI3K. [file 12894_2021_935_MOESM11_ESM.pdf]

Figure S12. PI3K

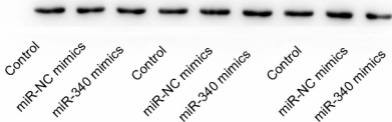

Supplement: Supplementary file 12 — Additional file 12. Fig. S12: PI3K. [file 12894_2021_935_MOESM12_ESM.pdf]

Figure S13. PCNA

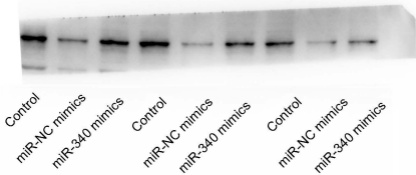

Supplement: Supplementary file 13 — Additional file 13. Fig. S13: PCNA. [file 12894_2021_935_MOESM13_ESM.pdf]
