# Supplementary material for: Overexpression of miR-340 inhibits cell proliferation and induces apoptosis of human bladder cancer via targeting Glut-1
Source: BMC Urol. 2021 Dec 3;21:168. doi: 10.1186/s12894-021-00935-z (PMC8641194; doi:10.1186/s12894-021-00935-z)
Supplement: Supplementary file 14 — Additional file 14. Table S1: Baseline characteristics. [file 12894_2021_935_MOESM14_ESM.docx]

**Table S1.** Baseline characteristics.

| **Character** |  |
| --- | --- |
| **Total, N** | 30 |
| **Age, Median (Range)** | 71 (43-94) |
| **Sex, N (%)** |  |
| Male | 20 (67%) |
| Female | 10 (33%) |
| **Cancer Type, N (%)** |  |
| Muscle-invasive bladder cancer (MIBC) | 16 (53%) |
| Non-muscle-invasive bladder cancer (NMIBC) | 14 (47%) |
| **Surgery, N (%)** |  |
| Radical Cystectomy | 12 (40%) |
| Trans urethral resection of bladder tumor (TURBT) | 18 (60%) |
| **Stage, N (%)** |  |
| I | 15 (50%) |
| II | 9 (30%) |
| III | 3 (10%) |
| IV | 3 (10%) |
